# Barth Syndrome: *TAFAZZIN* Gene, Cardiologic Aspects, and Mitochondrial Studies—A Comprehensive Narrative Review

**DOI:** 10.3390/genes16040465

**Published:** 2025-04-18

**Authors:** Consolato M. Sergi

**Affiliations:** 1 AP Division, Pathology Laboratories, Children’s Hospital of Eastern Ontario, University of Ottawa, 401 Smyth Rd., Ottawa, ON K1H 8L1, Canada; csergi@cheo.on.ca; Tel.: +1-613-737-7600; Fax: +1-613-738-4837; 2 Department of Laboratory Medicine and Pathology, University of Alberta, Edmonton, AB T6G 2R3, Canada

**Keywords:** Barth syndrome, BTHS, *TAFAZZIN*, TAFAZZIN, *TAZ*, TAZ, cardiovascular disease, left ventricular non-compaction, metabolic disease, cardiac surgery, prognosis, outcome

## Abstract

Barth syndrome (BTHS) is inherited through an X-linked pattern. The gene is located on Xq28. Male individuals who inherit the *TAFAZZIN* pathogenic variant will have the associated condition, while female individuals who inherit the *TAFAZZIN* pathogenic variant generally do not experience the condition. There are several organs that may be affected, but striking is the cardiological involvement. Cardiovascular disease, which may be the trigger starting the diagnostic procedure in a proband, may include a range of diseases from a severely dilated heart to a hypertrophic heart in the spectrum of anomalies encountered. Left ventricular non-compaction of the heart is also occasionally encountered. This cardiac event may reveal the prognosis of the affected patients. In this narrative review, we highlight the gene’s characteristics, the reactome, the cardiological features of the cardiovascular disease observed in patients affected with BTHS, emphasize the most current studies on BTHS cardiomyopathy, and delineate the biological underlying mechanisms supporting the proposal of new therapeutic options.

## 1. Introduction

Barth syndrome (BTHS) is identified in males who have specific symptoms, including cardiomyopathy, neutropenia, muscle weakness, delayed growth before puberty, and unique facial appearance [1,2,3]. The gene underlying this disorder is *TAFAZZIN*, which leads to unusual cardiolipin (CL) metabolism on the inner mitochondrial membrane. Although *TAFAZZIN* is universally expressed, BTHS involves a complex combination of tissue-specific phenotypes. It is important to note that not all these symptoms may be present in every affected individual. BTHS cardiomyopathy, often exhibiting dilated cardiomyopathy with or without endocardial-based fibroelastosis or left ventricular non-compaction (LVNC), is virtually always evident before the age of five years. Hypertrophic cardiomyopathy may also occur and may complicate the clinical picture. Heart failure is a major contributor to illness and, probably, death. There is, indeed, an elevated risk of cardiac arrhythmia and sudden cardiac death among BTHS patients, but sepsis due to neutropenia is also a leading cause of death. Neutropenia is mostly linked to the occurrence of repeated infections, including oral ulcers, pneumonia, and bacteremia. Neutropenia in BTHS correlates with life-threatening infections; however, the molecular and physiological mechanisms underlying this condition remain poorly understood [4,5].

The non-progressive myopathy mostly affects the muscles around the center of the body, leading to early motor neuron deficiencies. Prepubertal growth delay is succeeded by a post-pubertal growth spurt characterized by significant “catch-up” growth [5,6,7,8,9]. Female individuals with heterozygosity and a normal karyotype display no symptoms and reveal normal results in biochemical testing. In this narrative review, we outline the *TAFAZZIN* gene, the cardiologic aspects of BTHS cardiomyopathy, the extra-cardiology findings of BTHS, and some therapeutic options and discuss the updated pre-transplantation guidelines.

## 2. Materials and Methods

This is a narrative review, and the PubMed and Scopus databases have been searched for the *TAFAZZIN* gene and BTHS since inception. A total of 787 articles were found (24 March 2025), and additional literature (5 items) arising from Google was added as grey literature. Gray literature encompasses information produced outside traditional commercial or academic publishing channels, including reports, conference proceedings, theses, and government documents. The more detailed breakdown of grey literature components includes the following items: (1) Reports: This includes various types of reports such as annual reports, research reports, technical reports, project reports, and evaluations. (2) Working Papers: These are preliminary or draft versions of research that may not yet be formally published. (3) Government Documents: This includes a wide range of materials produced by government agencies, such as policy documents, white papers (in-depth reports or guides that provide information on a specific topic or issue, often used for advocacy or to inform policy decisions), and technical documentation. (4) Conference Proceedings and Abstracts: Papers and abstracts presented at conferences are often considered grey literature, especially if they are not formally published in a journal. (5) Theses and Dissertations: Academic research in the form of theses and dissertations is often not widely distributed through traditional publishing channels. (6) Technical Reports: These often contain detailed technical information and findings from research projects or studies. (7) Patents: Information about inventions and innovations, often found in patent documents. (8) Unpublished Clinical Trials: Data and results from clinical trials that are not published in peer-reviewed journals. (9) Newsletters and other internal communications. (10) Blogs and Social Media Posts. (11) Market and Industry Research. (12) Standards and Guidelines.

Cohort studies only and no single case reports harboring BTHS were used to report anomalies identified in individuals affected by *TAFAZZIN* gene changes. The quality of the single papers was carried out using a standard ad hoc method. SPRING and REACTOME were used to generate the molecular interactions of TAFAZZIN protein with other molecules and the signaling pathways.

We carried out a narrative review instead of the systematic review. The main objective of a systematic review is to formulate a well-defined research question. In a systematic review, qualitative and quantitative methods are used to analyze all the available evidence attempting to answer the question. In contrast, narrative reviews can address one or more questions simultaneously with a much broader scope, which was our choice. The efficacy of narrative reviews is irreplaceable in tracking the development of a scientific principle or a clinical concept or reviewing a condition using broad scopes. This ability to conduct a wider exploration could be lost in the restrictive framework of a systematic review. While the PRISMA statement and guidelines are critical for systematic review, narrative reviews need to follow a specific approach (e.g., IMRAD). We indeed used the preferred approach of the IMRAD (Introduction, Methods, Results, and Discussion) protocol. Apart from the author’s preferences, a narrative review structure must respect the journal style and conventions followed in the respective field (e.g., pediatric cardiology).

## 3. Results

The results are organized in the subsequent sections, including the *TAFAZZIN* gene (formerly known as *TAZ*), the TAFAZZIN protein (formerly known as TAFAZZIN), the animal models, the reactome and connectome of TAFAZZIN, BTHS, BTHS epidemiology, clinical data on BTHS arising from the two cohort studies identified in the literature specifying BTHS cardiomyopathy and extra-cardiac symptomatology, differential diagnosis, and prognosis.

### 3.1. TAFAZZIN (TAZ) Gene

**Figure 1:**
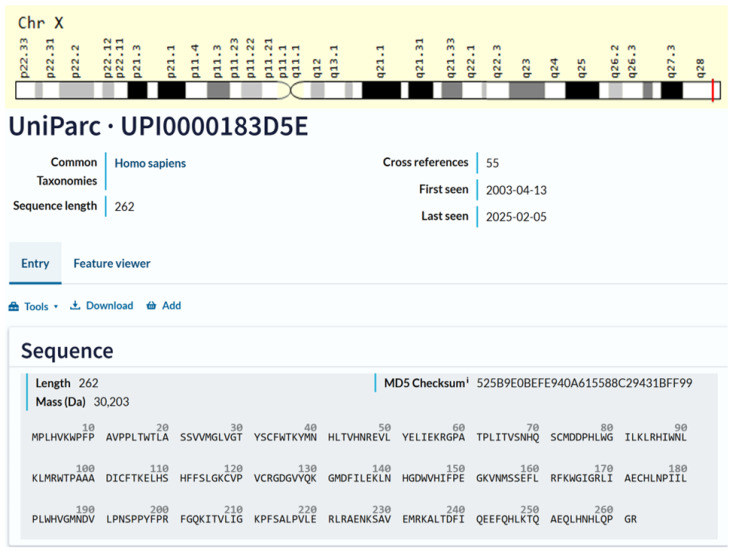
Cytogenetic localization and amino acid sequence of *TAFAZZIN*. Source: NCBI Bethesda, MD, USA, and GeneCards®: The GeneCards Suite: From Gene Data Mining to Disease Genome Sequence Analyses (PMID: 27322403) Stelzer G., Rosen R., Plaschkes I., Zimmerman S., Twik M., Fishilevich S., Iny Stein T., Nudel R., Lieder I., Mazor Y., Kaplan S., Dahary D., Warshawsky D., Guan-Golan Y., Kohn A., Rappaport N., Safran M., and Lancet D. Current Protocols in Bioinformatics(2016), 54: 1.30.1–1.30.33.doi: https://doi.org/10.1002/cpbi.5 “URL (accessed on 25 March 2025)”. Source: Universal Protein Resource (UniProt). The UniProt is a comprehensive resource for protein sequence and annotation data. The amino acid sequence is Q16635-3, which is the isoform that has been chosen as the canonical sequence. All positional information in this entry refers to it. This is also the sequence that appears in the downloadable versions of the entry. The UniProt Consortium, UniProt: The Universal Protein Knowledgebase in 2025 is reported in Nucleic Acids Res. 53: D609–D617 (2025). UniProt: https://www.uniprot.org/help/license “URL (accessed on 25 March 2025)”. UniProt has chosen to apply the Creative Commons Attribution 4.0 International (CC BY 4.0) License to all copyrightable parts of our databases. UniProt is a Global Biodata Coalition (GBC)-supported global core biodata resource. The GBC was created in 2020 as a consortium of international research funders dedicated to improve our understanding of the global biodata resource ecosystem The mission of UniProt is to provide the scientific community with a comprehensive, high-quality, and freely accessible resource of protein sequence and functional information.

The *TAFAZZIN* gene has eleven exons, and its 5-prime end corresponds to a CpG island, as found by Bione et al. (1996) [10]. The *TAFAZZIN* gene was located inside the crucial BTHS region on Xq28, according to Bione et al. (1996), who used positional cloning to confirm this [10]. In Figure 1, the location of the gene on chromosome Xq28 and the amino acid sequence of the gene are demonstrated.

**Figure 2:**
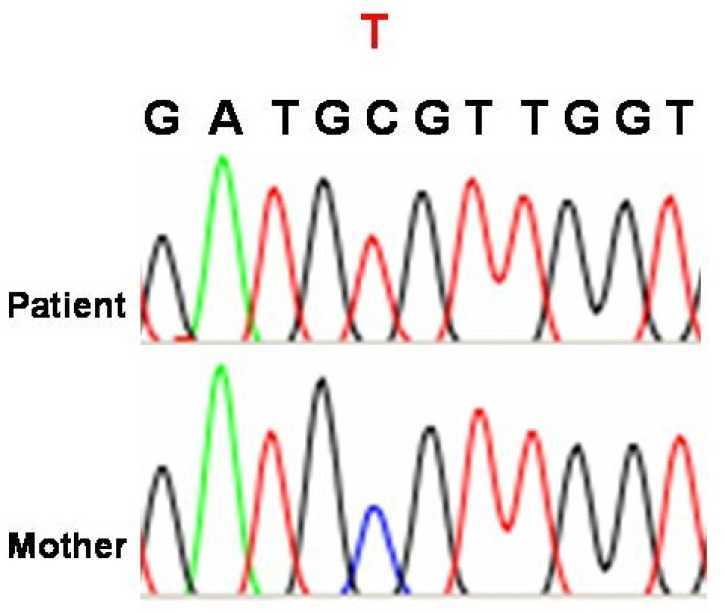
Mutation identified in a patient harboring BTHS. The DNA sequence of the *TAFAZZIN* gene on chromosome Xq28 shows a c.280C>T mutation (p.Arg94Cys), which leads to an amino acid exchange (courtesy of Professor Dr. MMAM Mannens, Dept. of Clinical Genetics, University of Amsterdam, Netherlands, to Dr. D. Karall and further courtesy to Dr. C. Sergi, email: 9 April 2025). Source: This case report was published in Karall, D. et al. (2014), Barth Syndrome and Left-Ventricular Non-Compaction: Case Report and Surveillance Plan Prior to Cardiac Transplantation. Enliven: Surgery and Transplantation. 01. 10.18650/2379-5719.12004 [22]. This is an open-access article published and distributed under the terms of the Creative Commons Attribution License, which permits unrestricted use, distribution, and reproduction in any medium, provided the original author and source are credited.

*TAFAZZIN* gene mutations may cause stop codons in the open reading frame, rendering most of the putative proteins incapable of being translated. Bione et al. (1996) discovered unique mutations in all their patients using mutation analysis on DNA samples from four families with BTHS [10]. In two of these family genes, linkage mapping was used in 1991 and 1993 [11,12]. In two smaller families with diagnostic features of the disease, exon 7, one of the alternative exons, was the site of the mutation [13,14]. In spite of predictions that the mutation would lead to truncation of the majority of TAFAZZIN proteins, a small number of variants deficient in exon 7 were found to be produced [13,14]. D’Adamo et al. (1997) found mutations in six out of eight extra probands with Barth syndrome when they examined the G4.5 gene (Xq28) [15]. Furthermore, D’Adamo et al. (1997) discovered a 1 bp deletion and a missense mutation in the G4.5 gene (Xq28) in three families with X-linked juvenile cardiomyopathy and/or endocardial fibroelastosis [15]. Although additional symptoms of BTHS could not be confirmed due to a lack of comprehensive clinical data on these patients, D’Adamo et al. (1997) proposed that G4.5 gene (Xq28) mutations could be a cause of infantile CMD in boys, even when no other symptoms of BTHS are present [15]. A new glycine-to-arginine substitution at position 197 was discovered when Bleyl et al. (1997) used single-strand conformation polymorphisms (SSCP) and DNA direct sequencing to scan the G4.5 gene for mutations in a family with non-compact texture of the left ventricular myocardium (LVNC) [16,17,18]. In their analysis of fourteen BTHS family trees, Johnston et al. (1997) discovered G4.5 gene (Xq28) mutations, five missense mutations, four splice site mutations, three deletions, one insertion, and one nonsense mutation [19]. Over the course of seven years, affected patients and obligatory carriers from five separate families with BTHS were studied at a National Health Service (NHS) hospital in Bristol, Southwest England, United Kingdom, by Cantlay et al. (1999), who also found mutations in the G4.5 gene (Xq28) [20]. It was recommended that all young male children with idiopathic dilated cardiomyopathy be evaluated for underlying BTHS, as the authors speculated that the condition might be more prevalent than initially thought. The G4.5 gene (Xq28) was examined by Chen et al. (2002) in 27 Japanese patients suffering from LVNC [21]. Of these patients, 14 were from familial instances within 10 families, and 13 were sporadic. A splice site mutation was found in one of these families. In Figure 2, a c.280C>T mutation (p.Arg94Cys), which leads to an amino acid exchange in the gene on chromosome Xq28, is demonstrated for one of our patients.

Currently, a genotype–phenotype heterogeneity is known for the patients studied in the literature.

### 3.2. TAFAZZIN (TAZ) Protein

Hypertrophic cardiomyopathy, dilated cardiomyopathy, endocardial fibroelastosis, and LVNC are among the clinical diseases of the heart linked to mutations in this gene. Claypool et al. (2006) demonstrated the 18-amino acid integral interfacial membrane anchor that localized yeast Tafazzin to both the inner and outer mitochondrial membranes [23]. This anchor integrated into the membranes but did not pass through the lipid bilayers. The Tafazzin from mice and human TAFAZZIN have many conserved or identical residues in this region. Yeast Tafazzin, an interfacial membrane anchor, had its membrane association changed due to mutations in conserved regions; these changes could have caused the protein to be mistargeted to the mitochondrial matrix or disrupted its assembly inside the mitochondrial membrane.

Various isoforms can be encoded by various transcript variations. Each of these isoforms has a long and short version, of which the short version does not have a hydrophobic leader sequence and might be found in the cytoplasm instead of attached to the cell membrane. The *TAFAZZIN* gene codes for a protein called TAFAZZIN. Dysregulation of the hippocampal merlin signaling pathway and glycerophospholipid production are two of its associated pathways. Acyltransferase is essential for the proper remodeling of the mitochondrial inner membrane phospholipid CL (1′,3′-bis-[1,2-diacyl-sn-glycero-3-phospho]-glycerol, or CL) with the specific acyl chains needed for proper mitochondrial function [24]. Improving mitochondrial functioning is its function in cellular physiology [25]. Lipid and protein co-assembly in mitochondria relies on CL. For example, remodeling of CL acyl groups in the mitochondrial inner membrane influences respiratory chain complex IV and its supercomplex forms’ assembly and stability. Tafazzin performs the transacylation of lysophospholipids to phosphatidylcholine at the highest rate of any phospholipid-to-lysophospholipid pair. The enzyme can catalyze a number of reactions, including the reacylation of 1-acyl-sn-glycero-3-phosphocholine, also known as lysophosphatidylcholine (LPC), and the transacylation of phatidylcholine (PC) to CL, which involves the exchange of acyl groups between the two compounds. It also lowers the rate of transacylations between PC and phosphatidate (1,2-diacyl-sn-glycero-3-phosphate, PA), phosphatidylethanolamine (1,2-diacyl-sn-glycero-3-phosphoethanolamine, PE), and CL, among other phospholipids.

As indicated above, Bione et al. (1996) found *TAFAZZIN* gene alterations in BYHS (BTHS; 302060) individuals [10]. High levels of expression of the gene, which they originally called G4.5, were observed in both cardiac and skeletal muscle. Proteins with distinct N-termini and core regions were encoded by alternate splicing of the main G4.5 transcript, which resulted in the production of distinct messenger RNAs. These proteins, which were named Tafazzins by Bione et al. (1996), are not related to any other proteins that are known to science. It is possible that two areas of the proteins have important functional roles. It is possible that the 30 residues at the N terminus, which are extremely hydrophobic, act as an anchor for the membrane. Tafazzins that are too short to contain the first two exons may be soluble cytoplasmic proteins without a hydrophobic region at their N terminus. The space in the middle, from amino acids 124 to 195, is the second region that can change. A hydrophilic region of the protein would gradually shrink upon deletion of exons 5, 6, and 7, perhaps creating an exposed loop that interacts with other proteins. As a result, the two most common variants differ in the hydrophilic and longest region expressed in exon 5. Reverse transcription polymerase chain reaction (RT-PCR) showed that several TAFAZZIN variants were expressed differently in different tissues, including fibroblasts, leukocytes, heart, and skeletal muscle [26,27,28,29].

### 3.3. Animal Models

Xu et al. (2006) found a loss of 80% in CL and diversification of its molecular makeup in homozygous *Drosophila* mutants that could not express full-length Tafazzin [30]. These findings are comparable to those described in individuals with BTHS. Reduced locomotor activity and frequent mitochondrial abnormalities, particularly in the cristae membranes, were seen in flies with the *TAFAZZIN* gene mutation, which affected their indirect flying muscles. In 2006, CL deficiency, caused by a lack of full-length Tafazzin, was a key component of the disease mechanism and resulted in mitochondrial myopathy [30,31]. Inducible short hairpin RNAs (shRNAs) can be used to knock down Tafazzin in mice starting from early embryos [32,33]. Tafazzin knockdown resulted in a significant drop in tetralinoleoyl CL concentration, along with an increase in mono-lyso-CLs (MLCL) and a change toward more saturated CL species. Skeletal muscle showed signs of ultrastructural anomalies at 2 months of age, while cardiac muscle showed signs at 8 months of age. In other tissues, there were no abnormalities found. Mitochondrial abnormalities encompassed alterations in mitochondrial architecture, vacuolization, mitophagy, and proliferation. Muscles that were affected also had myofibrillar disarray and enlarged vesicles that related to mitochondria and the endoplasmic reticulum. Damage to heart function was observed during Tafazzin knockdown, including left ventricular enlargement and muscle mass loss, decreased fractional shortening, and ejection fraction. In chronic CL insufficiency, damage of the mitochondrial architecture in sarcomeric tissues occurs over time [32,33]. In a separate study, Soustek et al. (2011) [34] used inducible shRNAs to suppress Tafazzin expression in mice; their results were comparable to Acehan et al. (2011) [32,33]. At 2 months of age, in the Soustek et al. model, the isometric contractile strength of the soleus muscle was diminished in Tafazzin-KO rodents [34]. Wang et al. (2023) compared the characteristics of first-generation (F1) Tafazzin KO/Y offspring to those of Tafazzin WT/Y littermates by crossing females from the C57BL6/J inbred strain with males from eight other inbred strains. Phenotypic expression was found to be significantly influenced by genetic background. The C57BL6/J and CAST/EiJ strains [F1] had, in contrast to A/J [F1] TafazzinKO/Y mice, which exhibited normal cardiac function, Tafazzin KO/Y mice that developed severe cardiomyopathy. Transcriptomic and metabolomic studies revealed indicators of mitochondrial decoupling and activation of the integrated stress response, and CL exhibited comparable defects in knockout mice across all genetic backgrounds. It seems that genetic modifiers change mitochondrial quality control and work downstream of CL to significantly affect the phenotypic expression of Tafazzin loss-of-function [35].

### 3.4. TAFAZZIN Reactome and Connectome

**Figure 3:**
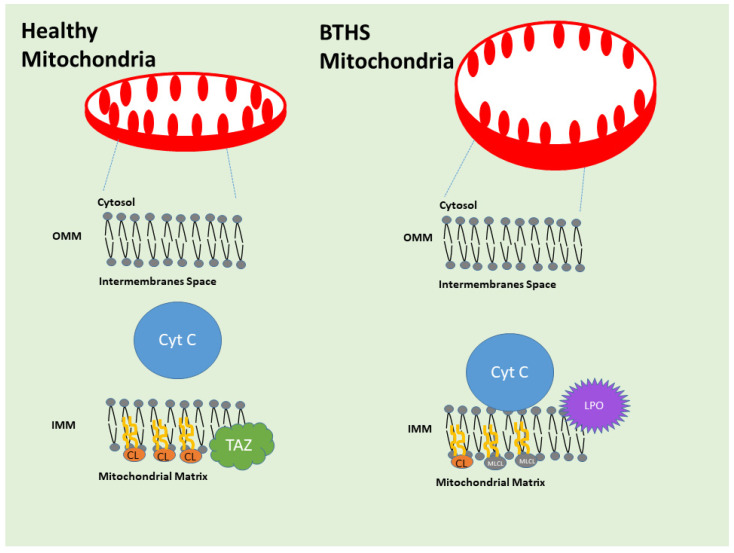
Tafazzin/TAFAZZIN in the mitochondrial inner membrane. Cardiolipin (CL) and 1-acyl lysophosphatidylethanolamine (LPE) are converted to monolysocardiolipin (MLCL) and phosphatidylethanolamine (PE), respectively, at the inner mitochondrial membrane (IM). The enzyme TAFAZZIN plays a critical role in cells by ensuring that the mitochondrial membranes incorporate the proper form of CL. In addition to providing structural support for the mitochondria, the membranes of these organelles also serve as a platform for a wide variety of critical chemical reactions, including those that generate cellular energy. CL levels drop, and levels of a precursor to it, mono-lyso-CL, or MLCL, rise in the setting of a *TAFAZZIN* gene mutation. Although scientists are aware that high concentrations of the precursor MLCL damage mitochondria, the precise mechanisms that lead from A (defunct TAFAZZIN) to B (BTHS symptoms) remain unclear. This extensive and persuasive study demonstrates that an excess of the precursor MLCL causes it to build up on the lipid membrane’s side, where it is in close contact with a protein known as cytochrome c. Cytochrome c undergoes a conformational and behavioral shift upon interaction with MLCL, enabling it to undergo a peroxidase transformation that results in the formation of hazardous phospholipids. The mitochondrial lipids are damaged when the precursor MLCL, mature CL, and other phospholipids undergo peroxidation. Experiments on yeast cells deficient in Tafazzin, mouse cells with reduced or absent Tafazzin, cells isolated from individuals suffering from BTHS, cardiac tissue from those individuals with mutated TAFAZZIN, and a fruit fly model of BTHS demonstrated the conversion of cytochrome c to a peroxidase following interaction with MLCL. Researchers demonstrated in both cell and fruit fly studies that the chemical imidazole oleic acid inhibits cytochrome c peroxidase activity and, in fruit flies, protects against exhaustion brought on by TAFAZZIN depletion. When TAFAZZIN is lost, the precursor to CL, MLCL, builds up to toxic levels. This causes the mitochondrial lipid membrane to become misorganized and causes MLCL to interact abnormally with cytochrome c. Cytochrome c produces harmful lipid byproducts because of this interaction, which in turn reduces energy generation and causes other mitochondrial dysfunctions. Source: Figure adapted and modified from Kagan et al. [40] (Kagan, V.E., Tyurina, Y.Y., Mikulska-Ruminska, K. et al. Anomalous peroxidase activity of cytochrome c is the primary pathogenic target in Barth syndrome. Nat Metab 5, 2184–2205 (2023). https://doi.org/10.1038/s42255-023-00926-4). The photograph was created using Microsoft PowerPoint, Windows 11, 2025 Update.

TAFAZZIN is located in the inner mitochondrial membrane in the cells (Figure 3). Xu et al. (2003), Xu et al. (2006), and Malhotra et al. (2009) found that Tafazzin converts CL and 1-acyl lysophosphatidylethanolamine (LPE) to MLCL and PE, respectively, at the inner mitochondrial membrane of the cell [30,31,36,37,38]. This molecule is not unique to any one region, but it does transfer acyl groups into the sn-1 and sn-2 locations of the MLCL, ensuring that the distribution of CL acyls is uniform and symmetrical. Because it is unable to transacylate dilyso-CL (DLCL), MLCL can only serve as an acyl acceptor. Not dependent on coenzyme A, it can rearrange molecular species within the same family of phospholipids. TAFAZZIN changes the ratio of fatty acids in MLCL to those in CL and other lipids, making CL’s half-life longer. CL performs a crucial function in mitochondrial membrane dynamics by adding to the lipid composition of the cell’s flexibility. Moreover, it is crucial for the last step of spermatogenesis, which is the differentiation of spermatids according to their similarities. It is necessary for spermatocytes to continue through meiosis. Catalysis requires human TAFAZZIN exon 7. Phosphatidylglycerol (1,2-diacyl-sn-glycero-3-phospho-(1′-sn-glycerol)) and lysophosphatidate (such as 1-acyl-sn-glycero-3-phosphate) are transacylated by the isoform 1 [39]. The third isoform of this protein is essential for the metabolism and remodeling of CL, and it also catalyzes the transacylation of lysophospholipids to cholesterol. Finally, mitophagy cannot begin without it [24]. A complex glycerophospholipid with four acyl groups, CL is involved in mitochondrial structure and function and is found localized to the inner membrane of mitochondria. In the Acehan et al. (2011) animal model, TAFAZZIN is a mitochondrial transacylase that helps transform immature CL into its mature form, which mostly contains tetralinoleoyl moieties [32,33].

**Figure 4:**
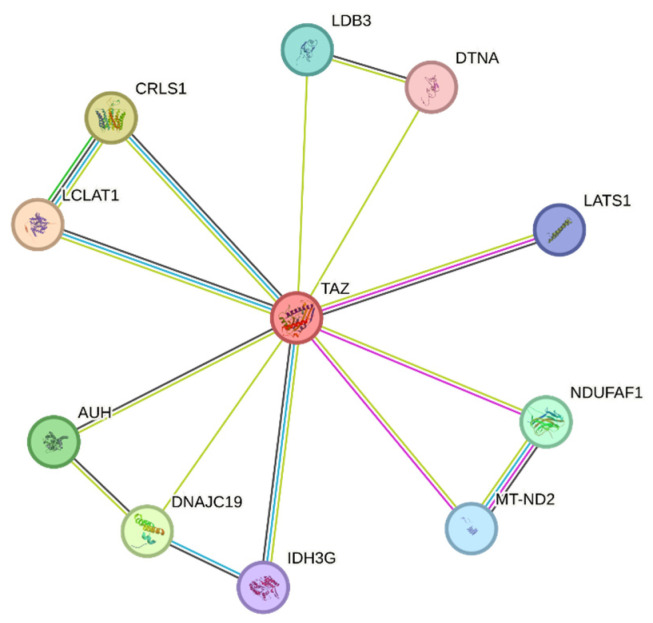
STRING-derived connectome of TAFAZZIN. Network nodes represent proteins. Splice isoforms or post-translational modifications are collapsed, i.e., each node represents all the proteins produced by a single, protein-coding gene locus. Colored nodes: query proteins and first shell of interactors; white nodes: second shell of interactors. Node Content: empty nodes represent proteins of unknown 3D structure, while filled nodes represent a 3D structure that is known or predicted. The edges represent protein–protein associations. Source: STRING Platform [41]. STRING is a database of known and predicted protein–protein interactions. The interactions include direct (physical) and indirect (functional) associations; they stem from computational prediction, from knowledge transfer between organisms, and from interactions aggregated from other (primary) databases. The STRING database currently covers 59′309′604 proteins from more than 10,000 organisms. All data and download files in STRING are freely available under a ‘Creative Commons BY 4.0’ license.

Other pathways where TAFAZZIN may play a role are under investigation, but glycerophospholipid biosynthesis, metabolism of lipids, mitochondrial protein import, and phospholipid metabolism are currently intensely studied. In Figure 4, the STRING-derived connectome of TAFAZZIN is demonstrated. Figure 4 has been generated using STRING software in our laboratories (Version 11, https://version11.string-db.org/ “URL accessed on 25 March 2025). It shows the connecting network of TAFAZZIN. STRING is a database of known and predicted protein–protein interactions. The interactions include direct (physical) and indirect (functional) associations. They originate from computational prediction, from knowledge transfer between organisms, and from interactions aggregated from other (primary) databases. STRING data sources include genomic context predictions, high-throughput lab experiments, (conserved) co-expression, automated text-mining, and previous knowledge in databases. The STRING database currently covers 59,309,604 proteins from 12,535 organisms (retrieved on 25 March 2025) (Szklarczyk et al. The STRING database in 2023: protein–protein association networks and functional enrichment analyses for any sequenced genome of interest. Nucleic Acids Res. 2023 Jan 6;51(D1): D638–D646. doi: https://doi.org/10.1093/nar/gkac1000. PMID: 36370105; PMCID: PMC9825434.) [41].

There is a protein called lysocardiolipin acyltransferase 1 (LCLAT1) that plays an important role in the remodeling of CL by catalyzing the reacylation of lyso-CL to CL. The Reactome shows that linoleoyl-CoA and oleoyl-CoA are the most suitable acyl donors for MLCL and di-lyso-CL, respectively, based on structural similarities. It also shows activity as an acyltransferase for 1-acyl-sn-glycerol-3-phosphate (AGPAT), which means it may change 1-acyl-sn-glycerol-3-phosphate (LPA) into 1,2-diacyl-sn-glycerol-3-phosphate (phosphatidic acid). The process of CL synthesis involves a specific transfer of a phosphatidyl group from CDP-diacylglycerol to phosphatidylglycerol, which is catalyzed by CL synthase (CRLS1). Whether the mitochondria are operating at peak efficiency or under extreme stress, CL, a critical phospholipid in mitochondrial membranes, keeps them intact and dynamic. One of the complexes needed for the ATP-dependent translocation of inner membrane proteins carrying transit peptides into the mitochondrial matrix is the Presequence Translocase-Associated Motor (PAM) complex, which is likely to include the mitochondrial import inner membrane translocase subunit translocase of the inner membrane 14 (TIM14). The ATP-dependent activity (similarity) could be stimulated by its potential role as a co-chaperone. Three hydroxy-3-methylglutaryl-CoAs are produced from three methylglutaconyl-CoAs by means of the mitochondrial methylglutaconyl-CoA hydratase. It converts itaconyl-CoA to citramalyl-CoA as part of the C5-dicarboxylate catabolism pathway; it also possesses itaconyl-CoA hydratase activity. Detoxifying itaconate, a compound that can harm people with vitamin B12, requires the C5-dicarboxylate catabolism route. Its enoyl-CoA hydratase activity is extremely poor. In vitro binding to clustered 5′-AUUUA-3′ motifs led to its initial identification as an RNA-binding protein. NADH dehydrogenase [ubiquinone] 1 alpha subcomplex assembly factor 1 (NDUFAF1) is a mitochondrial protein that is involved with complex I intermediates. In striated muscle, LIM domain-binding protein 3 (LDB3) may connect signals mediated by protein kinase C to the cytoskeleton through its LIM domains. The domain name is an acronym of the three genes in which it was first identified (*LIN-11*, *Isl-1* and *MEC-3*) Assumed to be a component of the minimal assembly necessary for catalysis, NADH-ubiquinone oxidoreductase chain 2 (MT-ND2) is a key member of the mitochondrial respiratory chain NADH dehydrogenase (Complex I). When electrons are transferred from NADH to the respiratory chain, the complex I is involved. Ubiquinone is thought to be the enzyme’s immediate electron acceptor based on similarities. A key player in the regulation of organ size and tumor suppression through the promotion of apoptosis and the restriction of proliferation, the serine/threonine-protein kinase LATS1 (Large tumor suppressor kinase 1) acts as a negative regulator of YAP1 (yes-associated protein 1) in the Hippo signaling pathway. YAP1 is a protein that acts as a transcription coregulator that promotes transcription of genes involved in cellular proliferation and suppressing apoptotic genes. At its heart, this pathway is a kinase cascade in which STK3/MST2 (Serine/Threonine Kinase 3/Macrophage Stimulating 2) and STK4/MST1 (Serine/Threonine Kinase 4/Macrophage Stimulating 1), in conjunction with their regulatory protein SAV1 (Salvador Homolog 1), phosphorylate and activate LATS1/2 (Large Tumor Suppressor Kinase 1/2), which in turn phosphorylate and inactivate YAP1 oncoprotein and WWTR1(WW Domain Containing Transcription Regulator 1)/TAFAZZIN, in complex with their regulatory protein MOB1 (Mps One Binder Homolog A). When LATS1 phosphorylates YAP1, it prevents the protein from entering the nucleus. Allosteric regulation of the enzyme catalyzing the decarboxylation of isocitrate (ICT) into α-ketoglutarate is facilitated by the mitochondrial subunit γ of isocitrate dehydrogenase (IDH3G), which is a regulatory subunit. While both the α (IDH3A) and β (IDH3B) and γ (IDH3G) heterodimers exhibit strong basal activity, the assembly and cooperative function of both heterodimers are necessary for the full activity of the heterotetramer, which contains two IDH3A subunits, one IDH3B subunit, and one IDH3G subunit. As a last point, dystrobrevin α (DTNA) could have a role in nicotinic acetylcholine receptor clustering, neuronal synaptic formation and stability, or both.

### 3.5. Barth Syndrome and Anatomic Pathology

**Figure 5:**
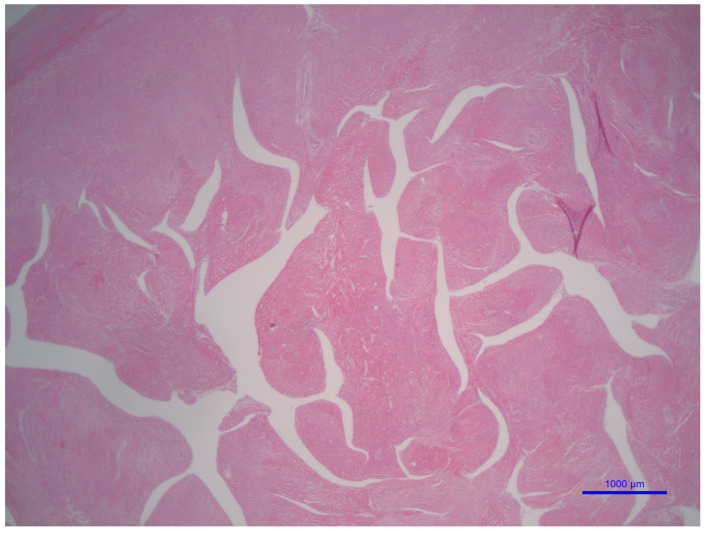
LVNC morphology in a patient with BTHS. This low-power microphotograph shows the trabeculation of the left ventricle using a transversal cut (hematoxylin and eosin staining, 2× original magnification, bar = 1 mm or 1000 μm). This microphotograph illustrates the left ventricle non-compaction histology of a patient affected with BTHS arises from the personal archive of the author (C. Sergi).

If a hemizygous pathogenic variation in the *TAFAZZIN* gene can be detected by molecular genetic testing or an elevated MLCL:CL ratio can be detected (if possible), then a male individual is proven to have BTHS [42,43]. Molecular genetic testing generally identifies a specific mutation known as a *TAFAZZIN* pathogenic variant in female patients exhibiting characteristic clinical symptoms, leading to a diagnosis of BTHS. To treat heart failure, one should pay close attention to fluid and volume levels, avoid dehydration and excessive diuresis, use the conventional drugs for heart failure to ease symptoms, and, in extreme and uncontrollable instances, consider a heart transplant. Antiarrhythmic drugs or implantable cardioverter defibrillators are potential treatments for cardiac arrhythmia. To treat neutropenia, a granulocyte colony-stimulating factor (GCSF) can be administered. Physical therapy may offer relief for weakness in the skeletal muscles. Conventional approaches can be used to treat scoliosis and talipes equinovarus, or clubfoot, which is a common foot anomaly in which the foot points downward and inward. A gastrostomy tube and feeding therapy may be necessary for patients with persistent feeding difficulties. Consumption of uncooked cornstarch before bedtime can help manage hypoglycemia. Common therapeutic approaches can help those with intellectual disabilities and developmental delays. Individuals with severe cardiac failure and/or considerable LVNC (Figure 5) should utilize continuous aspirin therapy to prevent blood clot formation and subsequent thromboembolic consequences.

Fully and well-investigated cohort studies are exclusively two reports, one from France and one from China (Table 1) [44,45]. In the French cohort, 14 patients exhibited cardiomyopathy, and 20 cases of cardiomyopathy were recorded during follow-up. The left ventricular systolic function was severely impaired throughout the first year of life and showed a tendency to normalize over time. French research discovered that 19 patients exhibited neutropenia. Metabolic analyses indicated variable moderate 3-methylglutaconic aciduria and plasma arginine concentrations that were diminished or within the low-normal spectrum. The Chinese cohort discovered *TAFAZZIN* gene mutations in four male index patients, including two unique variants (c.527A > G, p.H176R and c.134_136delinsCC, p.H45PfsX38). All four probands and two more afflicted male relatives were born at full term, with a median birth weight of 2350 g (range, 2000–2850 g). The median age at diagnosis of cardiomyopathy was 3.0 months (range, 1.0 to 20.0 months). The baseline echocardiogram demonstrated significant dilatation and trabeculations of the left ventricle, accompanied by compromised systolic function in six individuals, four of whom met the diagnostic criteria for LVNC. Additional elements of their clinical presentations were hypotonia (6/6), growth retardation (6/6), neutropenia (3/6), and 3-methylglutaconic aciduria (4/5) [45].

The best way to stop infections from happening again is to give people antibiotics before infections can start, but continuous use of antibiotics may trigger antibiotic resistance and jeopardize the gut and bile microbiome [46,47,48]. Restricted fasting or intravenous glucose infusion should be administered before scheduled procedures. When providing intravenous fluids containing potassium or during episodes of diarrhea, it is crucial to periodically monitor potassium levels [49]. Annual echocardiograms and electrocardiograms with Holter monitors are part of the surveillance process [5,18,50,51,52]. When a potentially life-threatening arrhythmia is suspected, an electrophysiologic investigation should be conducted. At the first sign of fever and twice annually thereafter, conduct a battery of blood tests, including a differential analysis. Checking for scoliosis, measuring the patient’s weight and height, and providing a clinical assessment of their strength should be carried out in patients affected with BTHS. Also, making notes of their educational needs and developmental milestones during each appointment should be critical for any pediatrician to monitor the development of a child affected with BTHS. Throughout a child’s formative years, a formal evaluation of their progress every three to five years should be considered mandatory. It is also critical to avoid agents or situations such as succinylcholine administration, rectal thermometer usage in neutropenic patients, and prolonged fasting [49]. Because most affected boys will reach a normal height by the time they enter puberty, it is usually recommended against giving them growth hormone unless there is clear proof of growth hormone insufficiency, in the current expert opinion [53]. In contrast to the general population, those with BTHS may be more likely to suffer from malignant hyperthermia because of muscle involvement. Identifying the relatives who may be in danger is also critical. A specialist obstetrician with experience in high-risk maternal–fetal care should oversee pregnancies with male babies diagnosed with BTHS. Each child born to a mother with a *TAFAZZIN* pathogenic mutation has a 50% chance of inheriting the mutation. The suffering man passes the *TAFAZZIN* pathogenic mutation on to all his female progeny but not to any of his male descendants. Prenatal testing can identify a higher-risk fetus, and testing for at-risk female relatives can be conducted if a *TAFAZZIN* pathogenic mutation has been found in an affected family member [1,54,55].

**Figure 6:**
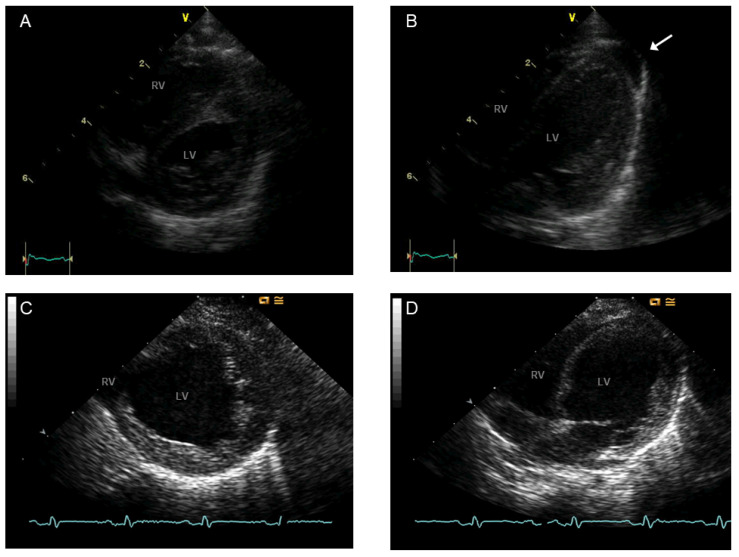
Echocardiography at the age of 2 weeks (**A**,**B**) and 8 months (**C**,**D**) of a patient with BTHS: Parasternal short axis view (left row) and atypical four-chamber view (right row). Normal systolic LV function (**A**,**B**) and minor pericardial effusion (arrow) at 2 weeks. Marked systolic dysfunction and LV dilatation at the age of 8 months, before heart transplantation (**C**,**D**). Abbreviations: RV, right ventricle; LV, left ventricle. Adapted from the following source: Karall et al. [22]. This is an open-access article published and distributed under the terms of the Creative Commons Attribution License, which permits unrestricted use, distribution, and reproduction in any medium, provided the original author and source are credited.

As indicated above, endocardial fibroelastosis and dilated cardiomyopathy are also possible diagnoses in BTHS in addition to LVNC. The ventricular chamber dilates and has trouble contracting, even though the left ventricle wall is normally thick and there may or may not be extensive thickening of the endocardium lining the ventricles. The medical problem known as LVNC occurs when the left ventricle of the heart does not compact adequately throughout development. Large trabeculations and deep intertrabecular recesses connect the left ventricular myocardium to the ventricular cavity (Figure 5). However, the finding of LVNC is identifiable not only at the autopsy table or operating room in the setting of a cardiac transplantation, but imaging guides the diagnostic approach precisely (Figure 6). If the left ventricle is not compacted, the diagnosis of hypertrophic cardiomyopathy should be ruled out by echocardiography and magnetic resonance imaging (MRI) [5,56,57]. A disorder called skeletal myopathy, or hypotonia, is defined by low muscle tone and weak muscles. Developmental delays preceding adolescence and macrocephaly (large head), full cheeks, a prominent pointed chin, large ears, and deeply set eyes are common dysmorphic traits seen in infants and toddlers.

Biochemically, patients with BTHS can disclose several pieces of evidence. One piece of evidence or validation from the laboratory is that (1) lactic acidosis typically occurs when blood lactic acid levels are higher than the normal range of 0.5–2.2 mmol/L. (2) A total cholesterol level below 110 mg/dL indicates hypocholesterolemia, which the subject possesses. (3) When the absolute neutrophil count is below 1500 cells per µL, it is referred to as neutropenia. (4) The organic acids in urine analysis revealed abnormally high concentrations of 2-ethylhydracrylic acid, 3-methylglutaric acid, and 3-methylglutaconic acid (3-MGC). (5) The usual increase in 3-MGC ranges from five to twenty times. With a standard deviation of µg/mg Cr, the average result is 44.6 ± 25 [58]. Keep in mind that a single urine sample may provide the impression that 3-MGC levels are within the normal range [59,60]. Also, keep in mind that these levels might be perfectly typical for the first six to eighteen months of a baby’s life [61]. An unusual biochemical result that points to a particular illness or condition is an increased ratio of MLCL to CL. People with BTHS show signs of cardiolipin remodeling insufficiency in multiple tissues because of a lack of protein in the inner mitochondrial membrane: CL levels, especially tetralinoleyl-CL, are low, while MLCL levels are high. 3-Methylglutaric acid content is moderately elevated. The content of 2-ethylhydracrylic acid has increased moderately, reaching a value of 14.4 ± 10.1. This growth is thought to be somewhat modest [62].

It is common for females with a *TAFAZZIN* mutation that differs from both parents to not exhibit any symptoms of BTHS. If a woman has exhibited symptoms of BTHS, there is usually a biological explanation for it, such as a ring X chromosome or X inactivation. A female carrying a structural X-chromosome aberration, such as a 45, X (Turner syndrome), may theoretically also have symptoms. A variety of molecular genetic testing methods are available, including but not limited to comprehensive genomic testing (e.g., genome sequencing, exome array, or single-gene testing) and gene-targeted testing (e.g., multigene panel testing). Characteristics or phenotypes are taken into account when deciding on the approach. While genomic testing does not demand that the clinician identify the exact gene(s) that are likely implicated, gene-targeted testing does. The vast variety of symptoms that can accompany BTHS makes gene-targeted testing the most likely method of diagnosis (Option 1) for those who display the classic symptoms. However, genomic testing is more likely to diagnose individuals whose symptoms are comparable to other genetic illnesses involving cardiomyopathy and/or hypotonia (Option 2). Genomic testing in which one gene is the primary focus. At the outset, *TAFAZZIN* is sequenced to detect changes at splice sites, missense, nonsense, and intragenic deletions/insertions. It is critical to keep in mind that different sequencing methods may have different capabilities when it comes to detecting deletions and duplications, particularly in females, whether they be single-exon, multiexon, or whole-gene. The next step, in the event that sequencing does not reveal any genetic changes, is to conduct gene-targeted deletion/duplication analysis in order to detect exon or gene deletions or duplications. Finding the exact genetic origin of the illness is best accomplished with a full panel of cardiomyopathy genes, which should include *TAFAZZIN* and other pertinent genes.

Exome arrays can identify deletions or duplications of numerous exons that sequence analysis misses; hence, they should be considered as a backup plan in case exome sequencing fails to produce a diagnosis. This method can only be used when the exome array is ready for clinical use. To the best of our knowledge and perusing the literature, there are more than 200 people known to have BTHS at this time [63].

Heart issues, especially dilated cardiomyopathy, usually manifest in infancy in most male individuals with BTHS. Researchers in France looked at 16 families that included 22 males with BTHS. According to Rigaud et al. [44], the participants in this study sought medical attention at an average age of 3.1 weeks, ranging from 0 to 1.4 years. A secondary analysis of 73 male participants in the BTHS Registry found that symptoms typically started at an average age of 0.76 ± 1.6 years, and the formal diagnosis was made at an average age of 4.04 ± 5.45 years [18]. The average duration between the onset of symptoms and the diagnosis of BTHS is, hence, around three years. For 73% of instances, the first sign was cardiomyopathy, while for 18%, it was infection. BTHS cardiomyopathy usually presents as dilated cardiomyopathy but can also show signs of hypertrophic cardiomyopathy alone or a combination of the two. Many affected men experience LVNC. Cardiomyopathy usually develops in a cyclical fashion, with heart tissue undergoing remodeling and going through phases of hypertrophy and dilation.

Diastolic dysfunction is the initial recognized biomechanical impairment in human hypertrophic cardiomyopathy, while systolic dysfunction is mostly the first sign of pathological physiology in dilated cardiomyopathy. On the other hand, hypertrophic cardiomyopathy variants produce hypercontractility-exhibiting, poorly relaxing sarcomeres via missense mutations in sarcomere proteins. Titin-truncating variants, yielding titin haploinsufficiency, are the most common causes of familial dilated cardiomyopathy [64].

In BTHS, the onset of cardiomyopathy usually occurs between the ages of one and five [58]. After the toddler years, some people with cardiomyopathy experience stabilization, while many others have improved. The size and mass of the left ventricle increased throughout the first six months of life, decreased until the age of two, and then stabilized [44]. However, insufficient data were found to adequately characterize these parameters in older children in our study. In their 2016 study, Kang et al. looked at 27 British individuals who had been diagnosed with BTHS. Longitudinal and circumferential strain abnormalities and reduced apical rotation were observed in persons with normal left ventricular size and function, according to the findings. This finding led to the suggestion that cardiac medications should be kept up for as long as mechanical or functional problems persist. Heart failure is a leading cause of death and sickness, and BTHS can cause variable degrees of cardiac function. Roberts et al. reports that there may be a gradual decline in heart function [18]. According to the BTHS Registry, the average ejection fraction z-score drops by 0.6 points for every five years of age increase. Out of 54 hospitalizations for heart failure, 11 were directly caused by infections that worsened the disease, according to the study by Rigaud et al. (2013) [44]. A total of two people in this group died from sepsis, while nine people died of heart failure. Between 1.2 and 30.7 months was the range of ages at which people passed away, with 5.1 months being the median. There was an observational study of 27 people in the UK registered with BTHS [51]. On average, seven of these people received heart transplants at a two-year interval, while five tragically did not make it past the average of 1.8 years. Cardiomyopathy or its harmful treatment-related complications was the exclusive cause of death. Medical management of heart failure usually results in a positive prognosis. Using standard cardiac medications to treat dilated cardiomyopathy, Spencer et al. [65] discovered that ejection fraction and left ventricular diastolic volume were normal in more than 16 of 30 males with the condition. However, other patients showed improvement at first with treatment, but then their health worsened after a while of stability, and they needed a heart transplant [66,67,68,69,70]. Heart rhythm problems. The risk of developing arrhythmia, such as ventricular and supraventricular tachycardia, and sudden death is increased. Children of any age can experience arrhythmia, although it is more typically seen in adolescents and young adults. Repolarization abnormalities and prolonged QTc intervals are common electrocardiogram (EKG) abnormalities. Twenty affected boys from the French cohort who had EKGs all showed a regular sinus rhythm. Repolarization abnormalities, including ST flattening and T-wave inversion, were observed in seventeen patients. The normal range for QTc is defined as QTc less than 420 ms, and five of the total participants had values within this range. In contrast, five people’s QTc levels were more than 460 ms. The QTc values ranged from 360 to 530 ms, with 440 ms being the median. In a study by Kang et al. [51], nine of the twenty-one patients had a QTc prolongation greater than 460 ms, and three had a borderline QTc prolongation between 450 and 460 ms. In five cases, ventricular arrhythmia led to cardiac arrest or the requirement for an internal defibrillator. The QTc intervals were all within the normal range in all five patients. There was a history of recurrent vasovagal symptoms in all five patients, including syncope upon shifting postures, nausea, and pale skin, suggesting autonomic instability. The left ventricle was slightly enlarged in one of the four people and slightly reduced in the other, both of which were within the usual range. Only one individual exhibited weak but persistent left ventricular function before suffering a cardiac arrest. Electrophysiological testing was able to cause ventricular arrhythmias in three patients. There was a history of sudden death in one family member who was thought to have BTHS, which affected two people and maybe a third. It should be noted that no associations were found between genetic variants and the probability of developing arrhythmia. A Holter monitor evaluation was performed on one subject, while another showed signs of repolarization issues at higher heart rates. Ventricular and supraventricular tachycardia were both felt by the patient. One of the two patients who had loop recorders implanted experienced a transient episode of atrial tachycardia. One patient had symptoms that were consistent with broad complex tachycardia, which was detected during the echocardiogram and lasted five beats. Sixty percent of people who participated in the BTHS Registry research had neutropenia. In one instance, 16 out of 22 males in the French study had a median absolute neutrophil count (ANC) below 500 cells/µL, which is similar to this percentage. Mild neutropenia is characterized by an ANC between 1000 and 1500 cells/µL. An ANC between 500 and 1000 cells/µL is considered moderate neutropenia. An ANC below 500 cells/µL is considered severe neutropenia. The median ANC may be about 1100 cells/µL [2], but ANC may be part of the group of congenital neutropenia syndromes, and BTHS needs to be well known to the neonatologist [71]. In a previous French cohort study, the median ANC ranged from 0 to 6400 cells/µL [44]. The ANC fluctuated across all examinations without any apparent pattern or regularity. Out of 88 people with BTHS, 84% had an ANC below 1500 cells per µL [49]. In their initial account of the disease, Barth et al. (1983) [72] noted that three out of seven men with a confirmed cause of death died of infection. The succeeding papers, however, failed to note any such alarming infection-related mortality. Recurrent oral infections are probably the most common symptom of neutropenia, while other symptoms may be milder. However, major issues might develop. Before G-CSF treatment was instituted, 35 BTHS patients were found to have infections, according to the UK NHS BTHS Service. In this group, two individuals had complications of acute tubular necrosis as a consequence of streptococcal septicemia; one needed a kidney transplant as a consequence of *haemophilus* septicemia; two individuals had osteomyelitis; one had septic arthritis; three individuals had soft tissue abscesses; five individuals had cellulitis; two individuals had balanitis; four individuals had lobar consolidation with lobar pneumonia; two individuals had gingivitis; and one individual had a urinary tract infection [49]. Oral ulcers affected 60.2% of men with BTHS, pneumonia affected 28.0%, and blood infections affected 10.0%, according to the most recent research on the BTHS Registry. A continual and substantial rise in monocyte levels could be the reason why bacterial infections happen so rarely, even though the ANC is always below 1000 cells/µL [58,62]. Out of 88 people diagnosed with BTHS, 75% had monocyte counts higher than 1000 cells/µL on at least one occasion [49]. Neutropenia in BTHS patients can be unpredictable and occur at random intervals, or it can be chronic and severe, or it can be cyclical and follow a predictable pattern [49].

Skeletal myopathy, which mostly affects the muscles around the center of the body, does not worsen with time [58]. It is common to diagnose affected children with hypotonia. Myopathy causes developmental motor delays, according to the BTHS Registry study. Forty-four of the sixty-seven children tested showed a delay in sitting up, and forty-eight delayed in walking. Notably, 34% of affected adults reported ever utilizing foot and ankle orthoses, walkers, or wheelchairs. Walking ages ranged from 12 to 24 months, with 19 months being the median. According to Adès et al. [11] and Gedeon et al. [73], hypotonia may manifest itself before birth in some males with BTHS who have talipes equinovarus. Most often, men with BTHS often have trouble exercising because their hearts do not work properly, and their muscles do not receive enough oxygen [65]. In a study comparing people with BTHS to a control group, researchers discovered that 33 out of 34 participants showed abnormal results in the six-minute walk test (6 MWT) distance [52]. In a 2019 study, 31 people diagnosed with BTHS had their functional exercise ability and strength tested and compared to the control group; individuals with BTHS performed worse on the 6-minute walk test (6 MWT), took longer to complete the five-times sit-to-stand test (5XSST), and exhibited lower knee extensor strength [74].

When compared to the typical curve, boys with BTHS exhibit a length that is approximately at the 50th percentile between the ages of six and thirty-six months. On the conventional curve, this would be about the 3rd percentile. Between the ages of 27 and 36 months, the average weight of these boys falls somewhere around the 3rd percentile on the standard curve [18]. There is a delayed puberty growth spurt, and then they show a lot of “catch-up” growth after that. The typical weight is somewhere around the 15th percentile.

A unique facial feature, especially apparent in infants, relates to BTHS in younger boys. Included in this description are features such as a high and broad forehead, a round face, full cheeks, a prominent pointed chin, big ears, and deeply set eyes. This physical trait is there throughout childhood; however, it starts to fade once a girl hits puberty. In addition to deep-set eyes, the ears are often somewhat large. Following the delayed puberty growth spurt, the distribution of fat in the gynoid region becomes the most noticeable feature at this stage [75,76].

BTHS is usually characterized by age-appropriate reading and language development in males. However, their visuospatial abilities are particularly lacking, and they often have below-average scores in math. These problems are not due to myopathy-related impaired motor function, as previously stated [77]. Problems with mathematics are not obvious in preschool but become apparent in kindergarten [78]. Thirty boys and men (aged three and up) were found to have delayed onset of language or difficulty forming sentences in the BTHS Registry study. There were 67 participants in all, and 31 of them had speech treatment. Of the forty-six boys older than seven years, twenty-two identified with a “learning disability” of some description. It is common to see feeding and eating issues in individuals who suffer from sensory impairments. People with this eating disorder tend to have a narrow dietary tolerance and a high preference for salty, cheesy, and spicy foods. Early on in life, issues including a strong gag reflex become noticeable [79]. A person’s psychological and social components interact to affect their overall health and capacity to participate in society; this is called psychosocial functioning. The quality of life for people with Barth syndrome is lower than that of healthy individuals and people with heart disease alone [80]. Of the thirty-four students, eight worked closely with a school counselor, and a school psychologist monitored nine. When a body function or system suddenly fails, it is called acute decompensation. Metabolic acidosis, elevated lactate levels, elevated transaminase levels, hypoglycemia, and elevated ammonia were the hallmarks of a severe metabolic disorder [75,81,82]. Delayed bone aging affects 58% of BTHS patients, while about 20% of cases exhibit scoliosis. Certain cardiac abnormalities, like endocardial fibroelastosis and subendocardial vacuolization of myocytes, may be associated with early male fetal loss due to BTHS. Some cardiac features may be detected as early as 18 weeks into pregnancy. Nine men out of sixty-five were born preterm between the 29-to-36-week gestational window, and nine out of forty-eight males had a birth weight less than 2.5 kg, according to the BTHS Registry study. The birth weight range in the French study was 2.18–3.73 kg, with a median of 2.77 kg. In addition, seven of the twenty-two boys in this study had severe intrauterine growth restriction (IUGR). The survival rate for males born before 2000 was 22%, but it jumped to 70% for those born in or after that year [44].

With regard to mortality and survival, the first is highest in the first four years of life, often due to heart failure or infections. While previously considered a lethal infantile and early childhood disease, improvements in management have led to better survival rates. Some patients are reported to live into their late forties, and a single patient even survived to age 51 [44]. However, the 5-year survival rate seems to be around 50% [44]. Nevertheless, it seems that the current survival rate should be quite high because there is better management of neutropenia and associated infections, improved treatment of cardiac disease, including systolic dysfunction and cardiac arrhythmias, and earlier and more accurate diagnoses.

Some of the test findings that could be associated with BTHS are as follows [83]. Plasma 3-MGC levels were greater in all 28 affected individuals in a single research study, which included subjects aged 10 months to 30 years. In a previous study, the range was from 393 to 2326 nmol/L, and the average level was 1088 nmol/L ± 435 [62]. In contrast, just eight individuals out of sixteen in the French group exhibited elevated 3-MGC levels [44]. The ratio between MLCL and CL is also a critical factor. The amounts of MLCL and CL in cultured fibroblasts from boys diagnosed with BTHS and a control group were quantified by Van Werkhoven et al. [84] using high-performance liquid chromatography–mass spectrometry (HPLC-MS). In the control group, the MLCL:CL ratios were between 0.03 and 0.12, whereas in the BTHS group, they varied from 5.41 to 13.83. Kulik et al. [85] used a bloodspot screening method to find that all patients with BTHS had a ratio of MLCL to CL more than 0.40, whereas all control subjects had a ratio lower than 0.23. If they used a threshold of 0.30, they found a 100% sensitivity and specificity. Classic BTHS is characterized by males with ratios greater than 1. People with an unusual or intermediate phenotype may have ratios that are less than 1 but still more than 0.4; they will have limited cardiac involvement, good exercise tolerance, and minor or no neutropenia. Diagnosis should be based on the MLCL:CL ratio rather than CL content alone to avoid false negative results for abnormal phenotypes that could be caused by assessing only tetralinoleoyl CL. This method has also been validated for use with lymphocytes, skeletal muscle, and cultured fibroblasts [86]. Characteristically, BTHS patients may show the overproduction of lactic acid, leading to acidosis. Depending on metabolic and cardiac issues, blood lactate levels might range from normal to substantially increased. For blood lactate, the usual range is between 0.5 and 2.2 mmol/L. The amino acids found in the liquid part of blood are called plasma amino acids. Eight men with a specific illness were the subjects of a French study that looked at their plasma amino acid levels. All these people had lower arginine levels than the control group, according to this study [44]. In a group of 28 men with BTHS, the previously indicated finding was confirmed; their average arginine level was 43 μmol/L, lower than the control group’s average of 70 μmol/L. The p-value that Vernon et al. [62] gave shows that this difference is statistically significant. The 28 men in this study showed significantly greater proline levels (291 μmol/L) than the control group (165 μmol/L). When blood cholesterol levels are below 110 mg/dL, it is known as hypocholesterolemia [87]. Although hypoglycemia is not commonly seen, it has been occasionally reported [44,88]. Moreover, the creatine kinase enzyme was elevated in 3 out of 20 BTHS patients, ranging from 192 to 397 mg/dL [87]. In the same study, fifteen of the nineteen males tested had prealbumin levels below 20 mg/dL [87]. In a separate study, Vernon et al. [62] found that lower levels of prealbumin were observed in 13 out of 18 affected men, with an average of 16.9 ± 4.0 mg/dL. In fibroblasts [89] and skeletal muscle [72], research into the respiratory chain shows impaired functioning of complexes III and IV. Mitochondrial abnormalities and lipid droplets in type I muscle fibers [72,90,91,92]. Orstavik et al. [93] found no symptoms in females who had one normal and one mutated *TAFAZZIN* gene on their active X chromosome. Two detrimental mutations in the *TAFAZZIN* gene were found in this individual. A ring X chromosome with a significant deletion contained the *TAFAZZIN* gene, which resulted in a full loss of the paternal allele as one mutation. A further mutation was identified as the deletion of exons 1–5 from the maternal *TAFAZZIN* gene [94]. Monosomy X with ring X chromosomal mosaicism was found during the investigation of lymphocyte and fibroblast cultures. It follows that her cells did not have a typical *TAFAZZIN* allele. The c.253insC (p.Arg85ProfsTer54) mutation in exon 3 was discovered in another female subject with a pathogenic *TAFAZZIN* variant. Hypotonia and left ventricular non-compaction were present in this patient. According to Avdjieva-Tzavella et al. [95], her blood tests showed that she had skewed X inactivation, which means that the nonmutated *TAFAZZIN* gene on her X chromosome was deliberately turned off. Studies conducted by Rigaud et al. [44] and Johnston et al. [19] show that genotype–phenotype relationships do not exist, as also reported above.

### 3.6. BTHS Cardiomyopathy

Individuals with BTHS are most often diagnosed and die from cardiomyopathy and heart failure [2]. Seventy percent of babies identified with BTHS had cardiomyopathy during the first year of life, and twelve percent required a heart transplant, according to 2012 data from the BTHS Registry [18]. The median age at transplant was 1.7 years, according to the most recent data [2], which are in line with the generally accepted belief that infants are at a higher risk than older children are. However, the underlying BTHS cardiomyopathy could be hard to diagnose because some concomitant viral infections cause symptoms that are comparable to myocarditis. Consequently, BTHS caused by metabolic or genetic causes may take longer to diagnose [2]. People who have BTHS often experience dilated cardiomyopathy, the most common type of cardiomyopathy. The ventricular walls thicken, and the heart muscle weakens, defining the condition. Furthermore, hypertrophic and restrictive cardiomyopathy are less prevalent in BTHS patients, and LVNC can occur in some patients [1,2,58]. Cardiac arrhythmias, prolonged corrected QT intervals, endocardial fibroelastosis, sudden cardiac arrest, and fetal cardiomyopathy with or without intrauterine fetal death are also possible cardiac disorders [1,2,58]. Biopsies of the heart taken from patients suffering from BTHS have shown myocyte hypertrophy, interstitial fibrosis, and endomyocardial fibroelastosis [1,2,58]. There were a lot of bigger, almost round mitochondria, as well as circular and tubular mitochondrial cristae, as shown by electron microscopy studies [96]. In addition, lipid microvesicles and vacuolated myocytes may be encountered in BTHS hearts [88].

Cardiomyopathy greatly affects the course and prognosis of BTHS [1,2]. Energy production, oxidative damage protection, membrane structure and organization, mitophagy, iron levels, and programmed cell death (apoptosis) are all regulated by CL, which plays an essential role in numerous mitochondrial processes [86,97,98]. Mitochondrial abnormalities can be caused by aberrant CL metabolism and malfunctioning TAFAZZIN. Mitochondria’s ability to generate ATP is facilitated by the respiratory chain that is housed within the inner mitochondrial membrane (IMM). The tricarboxylic acid (TCA) cycle (Krebs cycle, citric acid cycle) is involved in this process. Complexes I and II obtain electron transfers from NADH and FADH2 (reduced flavin adenine dinucleotide), respectively, generated by the TCA cycle, and cell lines derived from individuals with BTHS, including fibroblasts, lymphoblasts, and induced pluripotent stem cells (iPSCs), exhibit a reduced ability for mitochondrial respiration, according to investigations conducted thus far [1]. Newborn cardiomyocytes and mitochondria from Tafazzin KD(knockdown) hearts were found to have reduced energy production capabilities when fed pyruvate, palmitoylcarnitine, or succinate in the experiments carried out on Tafazzin KD animals [99,100]. As seen above, TAFAZZIN is an essential regulator of CL remodeling in mitochondrial respiration. Complex V, which is inserted into the IMM, completes the respiratory chain, which consists of four major protein complexes (I–IV) [101]. Cardiac tissues collected from BTHS patients showed decreased complex I activity [102]. Cardiomyocytes derived from BTHS patient iPSCs exhibited inefficient complex V activity due to a reduction in ATP generation [103]. Complexes I, II, III, IV, and/or V were all found to be underactive in the hearts of Tafazzin KD mice [1]. Efficient electron transport requires the assembly of respiratory chain complexes into large oligomers of varied stoichiometry and composition. Respiratory chain supercomplexes (RCS) are the name given to these oligomers [104]. Reactive oxygen species (ROS) are produced when complex I is assembled. This complex lays the groundwork for the attachment of complex III (III2), which is dimeric, and several occurrences of complex IV. The oxidation of NADH and FADH2, which are produced by the TCA cycle, into NAD+ and FAD+, respectively, occurs in mitochondrial oxidative phosphorylation (OXPHOS). The respiratory chain receives the electrons released during this oxidation. Low levels of NADH and FADH2 are maintained in a normal mitochondrion due to the respiratory chain’s excellent efficiency. Reduced respiratory capacity causes an increase in NADH buildup, which in turn inhibits TCA cycle enzyme activity [54,101,104,105,106]. Hearts of Tafazzin KD mice had far lower amounts of free CoA and acyl-CoA content than hearts of wild-type controls [99]. The finding that CL has a function in acetyl-CoA synthesis in yeast is in line with this finding [107,108]. Mitochondria serve as a significant origin of ROS, which are formed because of the electron transport activity occurring within the mitochondria. Elevated levels of ROS, such as superoxide, may originate from a decrease in the activity of respiratory chain complexes. Most studies conducted on BTHS iPSC-derived cardiomyocytes, Tafazzin KD, and KO cardiac mitochondria have reported elevated amounts of ROS [1,8,26,109,110,111,112]. Recent research by Bertero et al. [113] investigated the effects of Tafazzin KD on cardiac contractility and calcium transients, finding that, in comparison to wild-type cells, Tafazzin KD cardiomyocytes showed elevated fractional sarcomere shortening. The initial level of Ca^2+^ transient amplitude was unaltered in Tafazzin KD cardiomyocytes, but the rate of Ca^2+^ decay was increased [113]. Tafazzin KD showed less accumulation of diastolic Ca^2+^ when subjected to the β-adrenergic agonist isoproterenol with 5 Hz stimulation. Ca^2+^ in mitochondria is thought to control cardiac bioenergetics and function. The IMM contains the mitochondrial Ca^2+^ uniporter (MCU) complex, which is responsible for tightly controlling the uptake of Ca^2+^ by the mitochondria [114,115]. In lymphocytes and cardiac tissues isolated from individuals with BTHS, Ghosh et al. found that the quantity and functionality of the naturally occurring MCU were decreased [114,115]. In C2C12 cells that had Tafazzin knocked out (KO), they similarly saw a decrease in MCU abundance and activity. Using yeast as a surrogate, these authors demonstrated that CL is essential for calcium transport in mitochondria and for keeping MCU stable and active. Ghosh et al. recently discovered that cardiac tissues, lymphocytes from BTHS patients, and Tafazzin KO C2C12 cells all have reduced levels of mitochondrial calcium uptake proteins 1 and 2 (MICU1 and MICU2). On the other hand, there was no change in the amounts of EMRE and MCU regulator 1 (MCUR1) proteins [114,115]. When it comes to controlling cell death, mitochondria play a pivotal role. CL is brought out into the outer mitochondrial membrane (OMM) to facilitate the recruitment of multi-protein complexes by providing a binding platform. Apoptosis cannot occur without these complexes. A receptor for tBid recruitment to the mitochondrial membrane may be the externalized cytoplasmic loop. In order to permeabilize the OMM and activate Bax, it is also required. When CL levels are low, tBid has a far more difficult time attaching to mitochondria. On the OMM, CL is essential for caspase-8 recruitment, oligomerization, and activation [116,117]. Produced from BTHS and HeLa cells with TAFAZZIN depletion, lymphoblasts show resistance to apoptotic induction [13,24,29,118,119,120,121,122]. However, whether this is valid for living things is still debatable. It is still debatable whether TAFAZZIN-deficient cardiomyocytes undergo increased apoptosis [1]. Ultrastructurally, numerous spherical mitochondria are densely packed and situated among the contractile protein filaments in adult elongated cardiomyocytes [33,123,124,125,126]. Distinct mitochondrial characteristics, such as a complex double-membrane structure, specialized cristae production, continuous fusion and fission processes, and mitophagy, a quality control mechanism, are directly related to mitochondrial functions. To our knowledge, no studies have yet elucidated the precise mechanisms by which TAFAZZIN and CL remodeling modulate OPA1 (Optic Atrophy 1) and PHB (Prohibitin) regulation. OPA1 is a dynamin-related GTPase, a protein involved in mitochondrial dynamics and maintenance. Prohibitins are a family of highly conserved proteins that play crucial roles in mitochondrial functions and various cellular processes. The proper shape of mitochondria, which is essential for their proper functioning, is maintained through constant fusion and fission events. Beclin 1, an important regulator in autophagosome formation, and LC3, the mammalian counterpart of Atg8, are interacting with CL [7,69,111,127,128,129].

### 3.7. BTHS Epidemiology, Differential Diagnosis, and Prognosis

Worldwide, between 230 and 250 men have been identified with BTHS thus far, according to a 2020 study by Miller et al. [63]. From 1995 to 2008, the estimated prevalence of Barth syndrome in France was 1.5 cases per million births (95% CI: 0.2–2.3), while in the United States it ranged from 1:300,000 to 1:400,000 per year based on recent diagnoses. In Southwest England and South Wales, it was 1:140,000 per live birth [58]. In 2020, Miller et al. performed a Bayesian analysis and found that 1 male per million would have BTHS [63]. Articles addressing the frequency and incidence of cardiomyopathy and neutropenia included subgroups of patients with Barth syndrome, which is used to arrive at this estimate. In children suspected of having metabolic abnormalities at birth, increased concentrations of the branched-chain organic acid 3-MGC in the urine are a common finding. 3-MGC mediates the reaction between leucine and the mevalonate shunt pathway, which links mitochondrial acetyl-CoA metabolism with sterol production. 3-MGCA is the defining feature of the inborn metabolic error category [130]. Primarily affected are tissues with a higher need for oxidative metabolism, including the CNS, heart, and skeletal muscles. Along with *LDB3*, *ACTC1*, *MYH7*, *PRDM16*, *MIB1*, *TNNT2*, *TPM1*, and *MYBPC3* (OMIM PS604169), there are more genes linked to isolated cardiomyopathy with LVNC caused by pathogenic mutations. There is a lot of overlap in the physical traits of different phenotypes, making it hard to tell them apart according to their genes. However, a family history suggesting X-linked inheritance (i.e., diseased males related through unaffected females) can indicate the presence of BTHS, since none of these other genes are located on the X chromosome. In many cases, the best way to find the correct gene is to conduct molecular genetic testing using a panel of genes. Up to 28% of patients with Duchenne muscular dystrophy may experience skeletal myopathy and LVNC. Unlike BTHS, however, LVNC tends to worsen over time in Duchenne muscular dystrophy. When the number of white blood cells in the blood drops dangerously low, a condition known as neutropenia occurs. There is a wide variety of possible inherited reasons for isolated neutropenia, including some serious disorders. There is an autosomal dominant inheritance pattern for ELANE-related neutropenia. It has been associated with cyclic neutropenia and congenital neutropenia, two forms of neutropenia. The recurrent fever, sores in the mouth and throat (such as gingivitis, sinusitis, pharyngitis, and mouth ulcers), and swollen lymph nodes in the neck (cervical adenopathy) are hallmarks of these two major hematologic illnesses. An autosomal recessive disorder known as Kostmann syndrome (OMIM 610738) is inherited through the family tree and manifests at birth as a severe form of neutropenia. Several patients with Kostmann syndrome were found to have homozygous pathogenic mutations in the *HAX1* gene (which encodes HCLS1-associated protein X-1) [71,131,132,133,134,135].

If an individual is diagnosed with BTHS, it is recommended that he or she undergo the evaluations listed in the following Table 2 to fully understand the illness, its complications, and its requirements. In our experience, we came across the fact that psychological support for both children affected with BTHS, and their families is crucial to maintain the unity of the family and persevere in the healthcare endeavors this syndrome requires.

**Figure 7:**
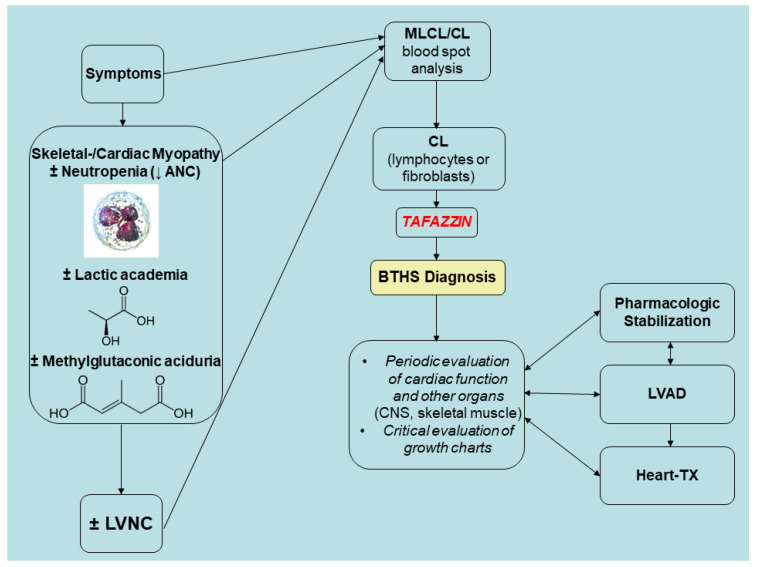
Pre-transplant strategic monitoring of BTHS patients. Adapted and updated (4 April 2025) from Karall et al. (2014), Barth Syndrome and Left-Ventricular Non-Compaction: Case Report and Surveillance Plan Prior to Cardiac Transplantation, Enliven: Surgery and Transplantation. 01. 10.18650/2379-5719.12004. Notes: CL, cardiolipin; LVNC, left ventricle non-compaction; MLCL, monolysocardiolipin; TX, transplantation. Source: [22]. This is an open-access article published and distributed under the terms of the Creative Commons Attribution License, which permits unrestricted use, distribution, and reproduction in any medium, provided the original author and source are credited.

Even on the first day of life, BTHS symptoms can manifest. In the past, infant and child mortality was high, and only a small percentage of patients made it until the fourth birthday. Infections caused by neutropenia and severe heart failure, which can cause metabolic decompensation and lactic acidosis, are the causes of death in BTHS. Nevertheless, fluctuating neutropenia, lactic acid, and 3-MGA concentrations are issues in BTHS patients [97]. In a metabolically stable time, a patient with BTHS may have normal values for all markers; nevertheless, in an agitated healthy patient, lactic acid concentrations might subsequently reach high levels, making diagnosis more challenging. According to the adapted Karall et al. [22] algorithm, the surveillance plan for patients with BTHS requires monitoring of lactic and 3-MGA as well as ANC. LVNC can be a marker of BTHS, but it is not obligatory, as seen above. Newborns with BTHS who are affected should undergo regular cardiac evaluations and undergo metabolic stabilization as soon as possible. Figure 7 proposes a strategy for monitoring BTHS patients prior to cardiac transplantation.

### 3.8. Future Directions

BTHS is an early-onset, lethal X-linked disorder caused by a mutation in *TAFAZZIN* gene, which encodes for a mitochondrial acyltransferase that remodels MLCL to mature CL and is essential for normal mitochondrial, cardiac, and skeletal muscle function. Current gene therapies in preclinical development require high levels of transduction. Most probably, *TAFAZZIN* gene therapy could be enhanced with the addition of a cell-penetrating peptide, penetrating (Antp). TAFAZZIN-Antp may be more effective than TAFAZZIN at preventing the development of pathological cardiac hypertrophy and heart failure.

### 3.9. Conclusive Remarks

BTHS cardiomyopathy is a unique pediatric cardiac disease caused by TAFAZZIN mutations affecting CL. Endomyocardial biopsy may show features that may be more substantive than the grossly identifiable LVNC. Monitoring before transplant needs to be tightened to avoid decompensation due to the still enigmatic role of CL and its role in autophagy, mitophagy, and programmed cell death. Multiple anomalies in CL profiles have been identified in patients with BTHS. Some of these issues include an inadequate concentration of CL, an imbalance in the fatty acyl chain composition of CL, and an excessive amount of MLCL relative to CL. Despite multiple studies that have demonstrated these irregularities, the exact role that each of these anomalies plays in the molecular deficiencies linked to BTHS is not easy to pin down. Additionally, whether TAFAZZIN may have non-cell–cell adhesion-dependent functions is still up in the air. Additional molecular biology and morphological research into the causes of BTHS cardiomyopathy will help us uncover the mysteries surrounding this condition and pave the way for more effective treatments.

## Figures and Tables

**Table 1:** French and Chinese cohort studies.

Country/#	Sex	Age (Y)	MofO	Genotype	ANC (×10^9^/L)	HF	SF/EF at DGN (%)	LVEDD z-Score at DGN	LV Mass z-Score at DGN	LVNC	SGA	Age of Walking (M)	3-MGCA	Informative CL Profile
France/1	F	0.09	CMP	Del exon 1-5	0.85	Yes	9/24	3.1	4.6	Yes	Yes	24	No	Yes
France/2	M	0.01	CMP	Exon 2/c.143delinsGG/p.Glu48fsX	1.93	Yes	20/38	4.5	−0.4	Yes	No	24	No	Yes
France/3	M	0.07	INF	Exon 3/c.280C > A/p.Arg94Ser	0.98	Yes	N.A.	0.7	1.4	No	No	20	Yes	Yes
France/4	M	1.37	CMP	Exon 3/c.281G > A/p.Arg94His	2.9	Yes	19/30	13	N.A.	No	No	N.A.	Yes	Yes
France/5	M	0.08	CMP	Exon 4/c.356T > G/p.Val119Gly	1.79	Yes	25/58	2.8	N.A.	Yes	No	N.A.	Yes	Yes
France/6	M	0.69	INF	Exon 6/c.478A > T/p. Lys160X	0.3	No	34/70	−0.7	−0.9	No	No	18	Yes	Yes
France/7	M	0.05	CMP	Del exon 6-11	0.77	Yes	14/N.A.	N.A.	N.A.	No	Yes	N.A.	Yes	Yes
France/8	M	0.11	CMP	Del exon 6-11	0.61	Yes	31/61	6.6	N.A.	No	Yes	18	Yes	N.A.
France/9	M	0.13	INF	Exon 8/c.589G > A/p.Gly197Arg	1	Yes	10/N.A.	6.6	N.A.	No	No	N.A.	N.A.	Yes
France/10	M	IU	CMP	Exon 8/c.589G > T/p.Gly197Trp	0.52	Yes	12.3/23.8	7.5	6.4	No	Yes	N.A.	Yes	Yes
France/11	M	IU	CMP	Exon 8/c.646G > A/p.Gly216Arg	13.63	Yes	25/N.A.	1.3	N.A.	No	Yes	N.A.	No	Yes
France/12	M	IU	CMP	N.T.	0.66	Yes	20/N.A.	1.3	N.A.	No	No	N.A.	N.A.	N.A.
France/13	M	0	CMP	Exon 8/c.646G > A/p.Gly216Arg	N.A.	Yes	N.A.	N.A.	N.A.	No	No	N.A.	N.A.	N.A.
France/14	M	0.1	INF	N.T.	0	No	N.A.	N.A.	N.A.	No	No	N.A.	N.A.	N.A.
France/15	M	0.71	CMP	Del exon 8-9	2.5	Yes	13.7/25.6	12.7	7.8	No	No	N.A.	N.A.	Yes
France/16	M	0.17	CMP	Exon 9/c.659_660dupGTCC/p.Leu221fsX	0.7	Yes	16/35	7.4	2	No	No	N.A.	No	Yes
France/17	M	0	CMP	Exon 9/c.659_660dupGTCC/p.Leu221fsX	1.47	Yes	30/N.A.	3.3	8.6	Yes	No	N.A.	No	Yes
France/18	M	1.7	FTT	Intron 9/c.700-1G > A/p.	0.72	Yes	8.5/16.3	8.4	3.5	Yes	Yes	N.A.	No	N.A.
France/19	M	0	Low Glu	N.D.	0.97	Yes	12.8/28.3	10.2	4.3	Yes	Yes	21	Yes	Yes
France/20	M	0	CMP	N.T.	2.88	Yes	N.A.	N.A.	N.A.	No	No	N.A.	N.A.	N.A.
France/21	M	0	CMP	Intron 10/c. 778-1G > T	3.82	Yes	16/36.1	1.9	3	Yes	No	18	Yes	Yes
France/22	M	0	CMP	Intron 10/c. 778-1G > T	4.28	Yes	N.A.	N.A.	N.A.	Yes	No	12	Yes	Yes
China/1	M	0.21	INF	c.527A > G (p.H176R)	N.A.	No	22.1/45.6	5.7	N.A.	Yes	N/A	N.A.	Yes	N.A.
China/2	M	0.21	HF	c.527A > G (p.H176R)	N.A.	Yes	16.7/36.2	3.8	N.A.	Yes	N/A	N.A.	Yes	N.A.
China/3	M	0.5	DMT	c.367C > T (p.R123X)	N.A.	No	19.1/40.1	3.3	N.A.	Yes	N/A	N.A.	Yes	N.A.
China/4	M	0.54	INF	c.710_711delTG (p.V237AfsX73)	N.A.	No	17.3/36.8	5.3	N.A.	Yes	N/A	N.A.	Yes	N.A.
China/5	M	0.08	INF	c.134_136delinsCC (p.H45PfsX38)	N.A.	No	18.9/40.1	5.7	N.A.	Yes	N/A	N.A.	No	N.A.
China/6	M	0.12	INF	N.D.	N.A.	No	20.0/43.0	4	N.A.	Yes	N/A	N.A.	N.D.	N.A.

Notes: French and Chinese cohorts compared (see text for details) [44,45]. 3-MGCA, 3-methylglutaconic aciduria; ANC, absolute neutrophile count; CMP, car-diomyopathy; DMT, decreased muscle tone, hypotonia; INF, infection; F, female; FTT, failure to thrive; HF, heart failure; Low Glu, hypoglycemia; LV, left ventricle; LVNC, left ventricle non-compaction; LVEDD-z score at DGN, z-score of left ventricular end of diastole diameter at time of the diagnosis; M, male; m, months; MofO, mode of onset; SF/EF at DGN, ratio between shortening fraction (SF) and ejection fraction (EF) at time of the diagnosis; SGA, small for gestational age; Y, years of age.

**Table 2:** Recommended assessments after initial diagnosis in males with BTHS.

Electrocardiogram (cardiac hypertrophy signs, QTc prolongation, and arrhythmia) Echocardiography to determine the size and non-compaction of the cardiac muscle Magnetic resonance imaging to evaluate cardiac stress and performance Immune system to grade neutropenia and immunologic competency Neurologic examination to detect degrees of hypotonia and signs of muscle weakness Measurement of the constitutional growth parameters (growth charts) Nutritional evaluation with a panel of experts in gastroenterology and nutrition Psychological assessment to incorporate cognitive, adaptive, and motor evaluations Assessment for eligibility for special education or early intervention programs Genetic counseling

Notes: Adapted and augmented recommendations on the basis of the literature and personal experience of the author as well as data from Roberts et al. (2012) [18].
